# The Effects of 24-Hour Neurosurgical Call on Fine Motor Dexterity, Cognition, and Mood

**DOI:** 10.7759/cureus.5687

**Published:** 2019-09-18

**Authors:** Cara M Rogers, Brian Saway, Christopher M Busch, Gary R Simonds

**Affiliations:** 1 Department of Surgery, Division of Neurological Surgery, Virginia Tech Carilion School of Medicine and Fralin Biomedical Research Institute, Roanoke, USA

**Keywords:** acgme duty hour restrictions, cognition, fine motor dexterity, mental well-being, neurosurgery

## Abstract

Background: Concerns regarding the effects of fatigue on physician performance and quality of life lead to the implementation of duty hour restrictions for residents by the Accreditation Council for Graduate Medical Education (ACGME). These restrictions have been met by strong criticism from the neurosurgical community. This is partly due to a lack of objective evidence that fatigue results in decrements in professional function in neurological surgeons. There is also concern that the restrictions have diminished clinical and operative experience as well as the development of professional responsibility in residency.

Objective: To evaluate whether 24-hour neurosurgical call has an objective impact on fine motor dexterity, cognitive thinking skills, and mental well-being.

Methods: Subjects were tested before and after taking 24 hours of neurosurgical call. We evaluated fine motor dexterity using the Vienna Test System Motor Performance Series, cognitive thinking abilities using a battery of paper-pencil neuropsychological tests, and mental well-being using the Profile of Mood States.

Results: A total of 27 subjects were included in this study, 12 seasoned to neurosurgical call and 15 naive to neurosurgical call. The seasoned subjects demonstrated no statistically significant change in performance after call on any of the tests for fine motor dexterity or cognitive thinking abilities. The nonseasoned subjects demonstrated multiple decrements in fine motor dexterity and cognitive thinking abilities after taking call. In the Motor Performance Series, they had a statistically significant decrease in the speed of untargeted movements in the nondominant hand during the tapping test (p = 0.002), and a decline in the precision of fine motor movements and information processing as evidenced by an increase in the number of errors of the dominant hand in the line tracking test (p = 0.014). There was a statistically significant decline in their immediate memory during Hopkins Verbal Learning Test (p = 0.025), and complex attention, mental flexibility, and visual-motor speed in the Trail Making Test (p = 0.03). The Profile of Mood States found no difference in feelings of anger (p = 0.54), tension (p = 0.358), or depression (p = 0.65). There were increased feelings of confusion (p < 0.001) and decreased feelings of vigor (p < 0.001) and friendliness (p = 0.001). Nonseasoned subjects had an increase in total mood disturbance (p = 0.012) but seasoned subjects did not (p = 0.083).

Conclusion: Our results suggest that fatigue-induced decrements in professional function can be ameliorated by experience with prolonged duty hours. In contrast to nonseasoned subjects, those who were conditioned to 24-hour neurosurgical call demonstrated resilience in fine motor dexterity and cognitive thinking skills, and exhibited no change in total mood disturbance. An argument can be made that we are turning the neurosurgical training paradigm upside down with the current ACGME duty hour restrictions.

## Introduction

Resident duty hours have been a topic of heated debate for the last 30 years and the 80-hour workweek limitation mandated by Accreditation Council for Graduate Medical Education (ACGME) has greatly impacted neurosurgical residencies [1-13]. Concerns for patient safety fueled this change with arguments that resident fatigue lead to greater errors in critical thinking and technical skills [3, 9-10, 14-17]. The rigorous work hours that were historically demanded during training were also felt to contribute to a decreased quality of life and physician burnout [3, 10, 16, 18]. However, now there is concern that these hour restrictions have limited educational opportunities that are critical to the training of a neurological surgeon [7, 11-13, 19]. Decreasing clinical and operative exposure could be having a deleterious impact on the maturation of critical thinking and technical skills that were felt to be endangered by fatigue [1, 5-6, 8, 12-13, 19]. Inadequate development of surgical and clinical decision making skills and a perceived failure of professional responsibility also have the potential to affect confidence and mental well-being [20]. It is therefore imperative that we determine whether extended work hours truly have a detrimental effect on physician performance, which would support the duty hour restrictions. The goal of our study was to evaluate whether 24-hour neurosurgical call has an impact on fine motor dexterity, cognitive thinking skills, and mental well-being.

## Materials and methods

Instruments

The methods for this study were adapted from a previous study assessing the effects of OR noise on motor skills, cognition, and mood [21]. We utilized the MLS Motor Performance Series of the Vienna Test Series by Schuhfried to assess fine motor dexterity. This is a modular test that utilizes Edwin Fleishman’s factor analysis of manual dexterity. It consists of a panel with various contact surfaces and the subject uses a stylus to perform static and dynamic tasks. We tested each subject’s dominant and nondominant arm in the Steadiness, Aiming, Tapping, and Line Tracking tasks. These tests measure the accuracy and precision of movement, steadiness, finger dexterity, and speed of finger, wrist, and arm movements [22-23]. 

Cognitive thinking skills were evaluated using a battery of standardized paper-pencil neuropsychological tests that have undergone extensive validation studies. The battery consisted of Hopkins Verbal Learning Test-Revised (Johns Hopkins University, Baltimore, MD), Brief Visuospatial Memory Test-Revised (Psychological Assessment Resources, Inc., Lutz, FL), Trail Making Test Form B (Reitan Neuropsychological Laboratory, Tucson, AZ), Symbol Digit Modalities Test (Western Psychological Services, Los Angeles, CA), and the Stroop Test (Stoelting Company, Wood Dale, IL). Hopkins Verbal Learning Test was used to measure verbal learning and memory [24]. Brief Visuospatial Memory Test was used to assess visuospatial memory [25]. Trail Making Test was utilized to evaluate the speed of processing, mental flexibility, and executive function [26]. Symbol Digit Modalities Test gaged attention, visual scanning, tracking, and motor speed [27]. Stroop Test measured selective attention, cognitive flexibility, and processing speed [28]. Clinical validity and reliability have been well established for this battery of tests in the evaluation of cognitive thinking abilities. 

To evaluate for the effect on mental well-being we utilized the Profile of Mood States created by McNair et al. [29]. This is a self-reported inventory to assess transient and enduring mood changes. It consists of 65 items that describe emotional states and the subjects grade each on a five point Likert scale. The items are grouped into emotional states of anger and hostility, confusion and bewilderment, depression and dejection, tension and anxiety, vigor and activity, fatigue and inertia, and friendliness. This is a commonly used tool used by psychologists to measure psychological distress. 

Participants

We tested 27 subjects taking 24 hours of neurosurgical call. All subjects were required to remain awake for the entire 24 hours to ensure a level of relatively consistent fatigue. The subjects’ fine motor dexterity, cognitive thinking abilities, and mood state were tested before and after 24 hours of call. A statistician then analyzed the data for statistically significant changes in the cohort from pre-call to post-call performance. We also compared the pre-call to post-call performance of seasoned subjects to nonseasoned subjects. We considered seasoned subjects to be those who had prior experience and were conditioned to taking 24 hours of neurosurgical call. We considered nonseasoned subjects to be those who did not have prior experience with taking 24 hours of neurosurgical call. We tested 12 seasoned subjects, which included neurosurgical attending physicians, resident physicians, and advanced care practitioners. We tested 15 nonseasoned subjects including medical students and undergraduate students.

Statistical analysis 

The scores for each subtest were reported numerically and analyzed by a statistician to assess for statistically significant effects on performance. Paired sample t-tests were used to assess for statistically significant differences in the pre-call to post-call performances of the entire cohort. We also used paired sample t-tests to evaluate the 12 seasoned subjects and the 15 nonseasoned subjects independently. We then used independent sample t-tests to compare the effect on seasoned subjects versus nonseasoned subjects. The cutoff value for statistical significance was chosen to be 0.05 to ensure at least a 95% confidence interval in the results.

## Results

MLS Motor Performance Series 

The steadiness subtest measured a subject's ability to position the arm and hand precisely and hold this position for a prolonged period. It required subjects to insert the 2 mm tip of the stylus into a 5 mm hole on the platform and maintain this position for 32 seconds. Each time that the stylus touched the sides or bottom of the panel it was recorded as an error. Performance was measured as the number of errors. There was no statistically significant difference in performance of the dominant arm for all subjects (p = 0.8). There was a statistically significant improvement in the performance of the nondominant arm of subjects after 24 hours of call with 3.4 less errors on average (p = 0.032).

The aiming task is a measure of hand-eye coordination and precision of movements. It required the subject to tap a line of twenty 5 mm diameter copper discs lined 4 mm apart, in succession with the stylus as quickly and precisely as possible. Each time that the stylus touched outside of the copper discs, it was recorded as an error. Performance was measured as the number of errors and by the amount of time it took the subject to complete the task. There was no statistically significant change in the number of errors made by the dominant arm in all subjects (p = 0.202). There was a statistically significant improvement in the speed of the dominant arm after call in all subjects with an average decrease in time of 0.4 seconds (p = 0.037). There was no statistically significant change in performance of the nondominant arm with regard to errors (p = 0.826) or speed (p = 0.106).

The tapping subtest measures wrist-finger speed and the speed of untargeted movements. The subject is instructed to tap the stylus against a square metal plate as rapidly as possible for 32 seconds. Performance was measured as the number of taps recorded by the panel in this time period. There was no statistically significant difference in the performance of seasoned subjects in the dominant hand (p = 0.603) or nondominant hand (p = 0.461). There was no statistically significant difference in the performance of the dominant arm in nonseasoned subjects (p = 0.772). There was a decrease in the speed of tapping after 24 hours of call in the nondominant arm of nonseasoned subjects measured as an average decrease in the number of hits by 7.9 (p = 0.002). 

The line tracking subtest evaluated the precision of arm-hand movements and information processing. This task required the subject to insert the stylus into a channeled maze that was 5 mm in width, and proceed through it without touching the sides or bottom of the panel. Each time that the stylus touched the sides or bottom of the panel it was recorded as an error. Performance was measured by the number of errors and the time it took to complete the maze. Seasoned subjects demonstrated no statistically significant difference in error rate of their dominant hand (p = 0.588) or nondominant hand (p = 0.289). Seasoned subjects demonstrated no statistically significant difference in speed of their dominant hand (p = 0.474) or nondominant hand (p = 0.710). Nonseasoned subjects demonstrated no statistically significant difference in performance of their nondominant hand with regard to errors (p = 0.772) or speed (p = 0.217). There was a statistically significant increase in the number of errors made by the nonseasoned subjects dominant hand after 24 hours of call with an average increase in errors of 4.4 (p = 0.014) but no statistically significant difference in speed (p = 0.98). 

Paper-Pencil Neuropsychological Tests

Hopkins Verbal Learning Test is a measure of verbal learning, immediate memory, and delayed memory [24]. Subjects were read a list of 12 words consisting of four words from three semantic categories. They were instructed to immediately recall as many of the words as they could in any order. This immediate recall test was repeated three times as trial 1-3. Trial 4 was completed after the other neuropsychological tests in which the subject was instructed to recall as many of the words from the original list to assess delayed memory. Subjects were given one point for each word recalled during the immediate and delayed recall trials. Immediate recall ability was measured as the sum of trials 1, 2, and 3. There was no statistically significant change in immediate recall ability of seasoned subjects (p = 0.135). There was a statistically significant decline in immediate recall ability of nonseasoned subjects after 24 hours of call with an average decrease of 2.4 words recalled (p = 0.025). Learning ability was measured by the higher of trial 2 or 3 minus trial 1. Retention ability was measured by the higher of trial 2 or 3 minus trial 4 multiplied by 100. There was no statistically significant change in learning ability (p = 0.663) or retention (p = 0.123) for all subjects.

The Brief Visuospatial Memory Test is a measure of visuospatial memory [25]. It consists of a page of six simple figures arranged in two columns and three rows. The subject is allowed to study the page for 10 seconds and is then instructed to draw the figures on a blank sheet of paper. They are given one point for each figure they draw correctly and another point if the location on the page is correct for a maximum score of 12. There was no statistically significant difference in performance after 24 hours of call in seasoned (p = 0.86) or nonseasoned subjects (p = 0.846). 

The Trail Making Test is a measure of complex attention, mental flexibility, and visual-motor speed [26]. The subjects are given a sheet of paper with 25 randomly arranged circles containing the numbers 1-13 and the letters A-L. They are given a pen and instructed to connect numbers to letters in ascending order. Performance is measured by the speed with which they complete the task. There was no statistically significant change in performance in seasoned subjects (p = 0.385). There was a statistically significant decrement in performance in the nonseasoned subjects taking an average of 5.6 seconds longer after 24 hours of call (p = 0.03).

The Symbol Digit Modality Test is a measure of psychomotor speed, short-term memory attention, and concentration [27]. It involves a simple substitution task in which subjects are given a reference key and required to pair numbers with their respective geometric figures. Performance was graded by the number of geometric figures they are able to convert into numbers in 90 seconds. There was no statistically significant difference in performance in seasoned subjects (p = 0.666) or nonseasoned subjects (p = 0.718). 

The Stroop Test is a test of cognitive flexibility and response inhibition [28]. Subjects are given a sheet of paper with five columns of 20 words. The words consist of the red, green, and blue but the written color is different than the printed color. The subjects are instructed to read out loud the printed colors and not the written words. Their performance is measured as the number of words they read out loud correctly in 45 seconds. There was no statistically significant difference in performance in seasoned subjects (p = 0.918) or nonseasoned subjects (p = 0.13).

Profile of Mood States

The Profile of Mood States was used to evaluate for changes in mood or psychological distress after 24 hours of neurosurgical call [18, 29]. The subjects were given a list of 65 words or statements that described feelings and asked to grade each based on how they had felt over the last week. They used a 5-point Likert scale ranging from 0 for feelings they had not experienced, to 5 for extreme feelings. The scores were grouped into mood categories including: anger and hostility, confusion and bewilderment, depression and dejection, tension and anxiety, vigor and activity, fatigue and inertia, and friendliness. 

There was no statistically significant change in feelings of anger and hostility (all subject p = 0.054, seasoned subjects p = 0.204, nonseasoned subjects p = 0.149), tension and anxiety (all subject p = 0.358, seasoned subjects p = 0.494, nonseasoned subjects p = 0.109), or depression and dejection (all subject p = 0.65, seasoned subjects p = 0.648, nonseasoned subjects p = 0.848). Seasoned subjects did not have a statistically significant change in feelings of fatigue and inertia (p = 0.132). Nonseasoned subjects did have a statistically significant increase in feelings of fatigue and inertia with an average increase in score of 5.6 (p = 0.002). There was a statistically significant increase in feelings of confusion and bewilderment in all subjects with an average increase in score of 2.1 in seasoned subjects (p = 0.02) and 3.6 in nonseasoned subjects (p = 0.005). There was a statistically significant decrease in feelings of vigor and activity with an average decrease in score of 3.3 in seasoned subjects (p = 0.016) and 5 in nonseasoned subjects (p = 0.009). There was a statistically significant decrease in feelings of friendliness with an average decrease in score of 2.1 in seasoned subjects (p = 0.026) and 1.8 in nonseasoned subjects (p = 0.027).

Total mood disturbance is calculated by adding the scores for tension, depression, anger, fatigue, and confusion and then subtracting the score for vigor. There was no statistically significant change in the total mood disturbance of seasoned subjects (0.083). There was a statistically significant increase in the total mood disturbance of nonseasoned subjects with an average increase in score of 14.6 (p = 0.012). 

## Discussion

The ACGME instituted the 80-hour work week limitation in 2003 in response to decades of concern that fatigue secondary to prolonged work hours results in mistakes that harm patients [3, 9-10,16-17]. The death of 18-year-old Libby Zion in 1984 due to a fatal medication interaction provided the impetus for reform. The residents involved in her care had been in the tail end of a traditional 36-hour shift. While the grand jury did not indict the involved physicians on charges of murder, they scrutinized the hospital system for a lack of supervision of routinely fatigued physicians in training [15, 30]. 

The Institute of Medicine released “To Err Is Human” in 1999, which reported that preventable medical errors resulted in more than 1 million injuries and up to 98,000 deaths each year in the United States and cost $17-29 billion dollars per year [17]. Then JAMA published an article in 2000 that listed medical errors as the third leading cause of death in the United States accounting for 225,000 deaths per year [14]. 

This led to the ACGME instituting an 80-hour work week restriction in 2003. They subsequently limited interns from working more than 16-hour shifts in 2011. This has resulted in significant criticism by many physicians especially in the academic neurosurgical community [4, 6-8, 10-11, 13, 19]. Multiple studies have been conducted that have failed to show an objective benefit to patient care after the implementation of the duty hour restrictions [4, 8, 12]. Neurosurgery is a unique profession that requires surgeons to maintain intense physical and intellectual stamina to perform complicated and lengthy operations. Duty hour restrictions are felt to limit essential operative and perioperative experiences that are crucial to attaining technical and clinical mastery in the diverse scope of neurosurgical diseases. There is also concern that it impedes the development of professionalism and surgical ownership. This could in turn lead to less adequately prepared neurosurgeons which would have a potentially devastating impact on patient care. 

We believe that this study raises interesting questions about neurosurgical training with respect to the alleged detrimental effects of prolonged work hours. The results suggest that fatigue-induced decrements in professional function can be ameliorated by experience. If this is the case, an argument can be made that we are turning the training paradigm upside down with the current ACGME restrictions.

Our results showed that subjects conditioned to taking 24 hours of neurosurgical call had no statistically significant decrement in fine motor or cognitive thinking abilities. In contrast, subjects that were not conditioned to the demands and fatigue of 24-hour neurosurgical call demonstrated decrements in several tests of fine motor and cognitive abilities. In the MLS Motor Performance Series they had a decrease in the speed of untargeted movements, precision of fine motor movements, and information processing. In the standardized neuropsychological tests, they demonstrated a decline in tests for immediate memory, complex attention, mental flexibility, and visual-motor speed. 

The effect of fatigue from 24-hour neurosurgical call on mood showed that there was no difference in feelings of anger, tension, or depression. There were increased feelings of confusion and decreased feelings of vigor and friendliness. Nonseasoned subjects were the only ones who had increased feelings of fatigue and more importantly had a statistically significant increase in their total mood disturbance. 

Future studies may aim at identifying other factors other than training level that may mitigate the effects of sleep deprivation such as caffeine intake, amount of sleep the previous night, and average hours of sleep per week, and self reported hours of sleep desired. Additionally, it may be beneficial to assess the behaviors of individuals that were significantly impacted as well as participants who were minimally impacted to assess for any behavior patterns that correlate with the effects of sleep deprivation on fine motor movement, mood, and cognition. 

## Conclusions

We conclude that conditioning the mind and body to fatigue should be viewed as an essential aspect of neurosurgical training as it has been shown to prevent fatigue-induced decrements in professional function. Subjects that were accustomed to the physical and mental stresses of 24-hour neurosurgical call demonstrated resilience in their fine motor dexterity and cognitive thinking skills, and exhibited no change in their total mood disturbance. As long and arduous hours are a fact of life in a neurosurgical career, learning how to recognize and manage fatigue during training will hopefully improve physician resilience and therefore patient care.
